# No association between chronotype and cardiovascular response to a cognitive challenge in the morning using a Bayesian approach

**DOI:** 10.1016/j.nbscr.2025.100125

**Published:** 2025-05-09

**Authors:** Larissa N. Wüst, Christian Cajochen, Ruta Lasauskaite

**Affiliations:** a Centre for Chronobiology, Psychiatric Hospital of the University of Basel, Basel, Switzerland; b Research Cluster Molecular and Cognitive Neurosciences, University of Basel, Basel, Switzerland

**Keywords:** Mental effort, Cardiovascular, Chronotype, Melatonin onset, Sleep restriction, Alertness, Sleepiness

## Abstract

A chronotype is defined as a preference for certain behaviours (e.g., sleep and wake) to occur at specific times of day. It is therefore also temporally linked with cognitive performance across the day. In an exploratory analysis, we sought to find associations between chronotypes determined from self-reported habitual sleep timing and from salivary melatonin onset with mental effort during a 2-back working memory task. Mental effort was operationalized as sympathetic beta-adrenergic impact on the heart, which is best reflected by the cardiac pre-ejection period (PEP) and also influences systolic blood pressure (SBP). Each participant underwent two experimental sessions in the morning: once after sleeping for 8 h and once after sleeping for 5 h the night before. To determine the timing of evening melatonin onset, participants took saliva samples at hourly intervals at home in the evening, prior to their experimental sessions. Chronotypes were determined using reported sleep times from the Munich Chronotype Questionnaire and average melatonin onset during both sleep conditions. Based on this, participants were grouped into early, intermediate, or late types. Neither alertness (*BF*_*10*_ = 0.019), perceived task demand (*BF*_*10*_ = 0.008), nor SBP response (*BF*_*10*_ = 0.268) were credibly impacted by sleep-time derived chronotype, while the association with PEP response (*BF*_*10*_ = 0.631) during a cognitive challenge in the morning was inconclusive. Similarly, the timing of evening melatonin onset did not affect alertness (*BF*_*10*_ = 0.003), perceived task demand (*BF*_*10*_ = 0.006), or PEP or SBP response (PEP: *BF*_*10*_ = 0.232, SBP: *BF*_*10*_ = 0.263) during the cognitive challenge. Our data shows no impact of chronotypes on effort-related cardiovascular response during a cognitive challenge in the morning, which was scheduled according to habitual sleep times.

## Highlights

• Sleep timing associated with melatonin onset sampled with minimal instructions.
• Chronotypes did not differ in invested effort in morning cognitive challenge.
• Early types determined from melatonin onset showed better working memory performance.

## 1 Introduction

An extensive body of research has shown circadian rhythms in alertness and cognitive performance measures, i.e., peak alertness and performance in the morning and deterioration over the day (for review see Schmidt et al., 2007). Such circadian rhythms are also reflected in sleep timing and several biological markers, such as melatonin onset and core body temperature (for review see Reid, 2019). Individual circadian rhythms are also influenced by the individual's chronotype, that marks a person's preferred bed times (early or late) but also their optimal or preferred time for cognitive and physical performance (for review see Adan et al., 2012). While effects of circadian time and chronotypes on alertness and cognitive performance have been investigated extensively, knowledge about effects on mental effort remains sparse.

### 1.1 Mental effort and motivation intensity theory

Effort is defined as the mobilization of resources to carry out instrumental behavior (Gendolla and Wright, 2009). Brehm's motivation intensity theory (Brehm and Self, 1989) posits that effort should be proportional to experienced task demand, as long as success is possible and the required effort is justified. Combining motivation intensity theory with Obrist's active coping approach (Obrist, 1981), Rex Wright suggested that effort should manifest in the beta-adrenergic sympathetic activity of the autonomous nervous system impacting the heart (Wright, 1996). The most reliable non-invasive index of the beta-adrenergic sympathetic impact on the heart is the cardiac pre-ejection period (PEP, see Kelsey, 2012; Richter et al., 2008), the time interval between the onset of excitation of the left ventricle of the heart, indicated by the Q-point of the electrocardiogram (ECG), and the opening of the aortic valve, indicated by the B-point of the impedance cardiogram (ICG, Berntson et al., 2004). Systolic blood pressure (SBP) is also mainly determined by sympathetic activity and is therefore frequently used to assess sympathetic activity of the heart (Wright and Kirby, 2001), nevertheless, SBP is also influenced by ventricular resistance which might mask effects.

Previous research has identified several factors which impact effort-related cardiovascular response, such as self-reported insomnia (Schmidt et al., 2010) or fatigue (Mlynski et al., 2017). Individuals with high self-reported insomnia showed stronger SBP reactivity during a moderate cognitive challenge (Schmidt et al., 2010). Mlynski and colleagues showed a stronger heart rate (HR) response in highly fatigued participants during the first minute of a moderately difficult cognitive challenge (Mlynski et al., 2017). No study to date has investigated effects of sleep duration or fatigue on cardiac PEP.

### 1.2 Subjective chronotyping

Chronotypes indicate a preference for a certain time of day, which goes along with preferences for sleep timing and markers of the circadian clock. Chronotypes are often determined through self-report instruments such as the Munich Chronotype Questionnaire (MCTQ, Roenneberg et al., 2003) or the Morningness Eveningness Questionnaire (MEQ, Horne and Ostberg, 1976). The MCTQ assesses habitual sleep timing to determine the chronotype, while the MEQ assesses behavioral and sleep preferences. Chronotypes have been shown to be associated with alertness, cognitive performance, and even neural activity, i.e., the hemodynamic response during an inhibitory control task (Schmidt et al., 2012).

Previous research showed that later types felt more sleepy compared to earlier types in the morning (Mongrain et al., 2008; Natale and Cicogna, 1996) or even throughout the entire day (Maierova et al., 2016). However, effects of chronotypes on cognitive performance were not univocal throughout studies. Chronotypes were found to impact sustained attention (Lara et al., 2014; Martínez-Pérez et al., 2020), executive control tasks (Martínez-Pérez et al., 2020), or workplace simulating tasks (Horne et al., 1980). Other studies were not able to show an impact of chronotypes on sustained attention (Mongrain et al., 2008) or working memory (Schmidt et al., 2015). Importantly, Lara et al. (2014) and Martínez-Pérez et al. (2020) administered the cognitive tests at fixed times, while Mongrain et al. (2008) and Schmidt et al. (2015) adapted test times to preferred sleep schedules.

Carbajal et al. (2019) investigated self-reported morning types in two experimental sessions, once in the morning and once in the evening, and found that task difficulty appraisal and effort-related cardiovascular response differed between the experimental sessions. While the effort-related cardiovascular response increased with task difficulty in the morning, which is the optimal time for performance of morning types, it maintained a low level irrespective of task difficulty in the evening. The effects of difficulty and subsequently effort were only present in women, but could not be found in men (Carbajal et al., 2019). Overall, effects of chronotype on mental effort appear mixed, while research is sparse, yet. Generally, it remains unknown whether differences in cognitive performance between chronotypes occur due to neurophysiological phenomena or whether motivational aspects cause such differences in cognitive performance.

### 1.3 The timing of melatonin onset and chronotype

The onset of melatonin secretion under dim light, i.e., dim light melatonin onset (DLMO), is one of the most common biological markers of the circadian clock's phase. DLMO has been shown to be strongly correlated with chronotypes determined from questionnaires (Reiter et al., 2021). Nevertheless, for any given mid-sleep time from the MCTQ, DLMOs occurred within a 4-h window (Kantermann et al., 2015). DLMO was highly correlated with habitual sleep and wake times (Mongrain et al., 2004) and later DLMO has been found to be related to stronger eveningness (Kennaway, 2023). Even though DLMO was also associated with chronotypes based on questionnaires, concordance between chronotype categorizations was rather poor (Reiter et al., 2021). Furthermore, later DLMO was associated with higher sleepiness and poorer attentional performance in the morning after sleep restriction (Sletten et al., 2015).

### 1.4 Present research

Here, we present an exploratory analysis based on existing data (preliminary study in Wüst and Lasauskaite, 2024) to determine associations between sleep time-derived and melatonin onset-derived chronotypes with alertness and mental effort during a cognitive challenge in the morning. In the study, we assessed habitual sleep timing using the MCTQ and the timing of evening melatonin onset from saliva samples collected by participants at home and categorized participants into early, intermediate, and late chronotypes based on each of these measures. Mental effort was assessed through effort-related cardiovascular response, namely cardiac PEP and SBP reactivity. We expected that later chronotypes, derived from sleep time and from timing of melatonin onset, would feel less alert in the morning, resulting in higher perceived task demand. Following the assumptions of the motivation intensity theory, later types should mobilize more effort during a cognitive challenge compared to earlier types, while maintaining the same level of task performance. Primary analyses of our dataset did not reveal any significant differences in mental effort, reflected through cardiovascular reactivity, between a sleep-restricted and a well-rested condition nor between two different light intensity levels. For more information, please refer to the preliminary study presented in the supplementary material of Wüst and Lasauskaite (2024).

## 2 Method

### 2.1 Participants

Forty right-handed participants (age *M* = 24.9 years, *SD* = 4.1 years; 24 women, 16 men) were recruited via an online message board of the University of Basel. To be eligible for participation, participants needed to be between 18 and 35 years old, have a body weight of 30–155 kg, and body height between 120 and 230 cm (as required for use of the CardioScreen 1000 device). Exclusion criteria were acute or a history of cardiovascular disease, current intake of psychopharmacological medication, deficiency in color vision, correction of vision through glasses, non-removable nail make-up, and pregnancy. All participants received monetary remuneration (CHF 150, approx. USD 165).

### 2.2 Procedure

The experimental procedure was adapted from earlier studies (Lasauskaite et al., 2019, 2023; Lasauskaite and Cajochen, 2018) and approved by the local ethics committee (Ethikkomission Nordwestschweiz).

Participants underwent an initial screening via telephone to check eligibility for study participation and afterwards were invited for a screening visit. During the screening visit, participants were tested for color vision deficiency using the Ishihara test (Ishihara, 2016) and answered the Munich Chronotype Questionnaire (MCTQ, Roenneberg et al., 2003) to determine their habitual sleep times and chronotype. Furthermore, participants were equipped with a wrist-worn actimeter (ActTrust, Condor Instruments, São Paulo, Brazil) that recorded activity and light, and were instructed how to perform saliva sampling.

**Fig. 1:**
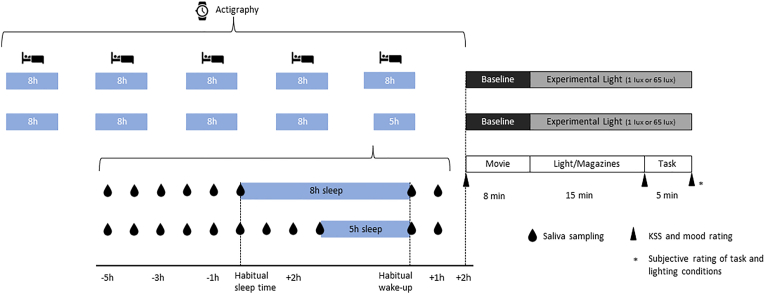
Study design. Actigraphy started 5 d prior to the experimental session to assess compliance with the given sleep-wake-schedule. Participants were collecting hourly saliva samples the evening before their scheduled experimental session, starting 5 h prior to their habitual sleep time until they went to sleep. In the morning, saliva samples were collected at wake-up and one hour later. Experimental sessions in the laboratory were scheduled two hours after habitual wake-up and consisted of a cardiovascular baseline period, a light exposure period and an auditory 2-back working memory task.

The experimental procedure is outlined in Fig. 1. Participants were instructed to adhere to a given rest-activity-schedule, based on their reported habitual sleep timing, with 8 h time in bed for four or five days, respectively (see below), prior to each of their experimental visits. These sleep-wake-schedules were for most participants closer to the required sleep-wake-schedules on work days than to preferred schedules on free days, as it was necessary to accommodate obligations of work schedules. During the days of the fixed rest-activity-schedule, participants filled in online sleep diaries, which were provided via the online tool REDCap (Harris et al., 2009, 2019) hosted at the University of Basel.

Each participant underwent two experimental visits, one after the last night before the experimental session with 8 h time in bed (normally rested; NR) and one after the last night with 5 h time in bed (sleep restriction; SR). Reduced time in bed was achieved by delaying bedtime for 3 h while keeping the wake time constant. Both sleep duration conditions were applied in a randomized, counterbalanced order (within participants).

Five hours prior to their habitual sleep time on the day before the experimental visits, participants started hourly saliva sampling. They noted the exact time of the saliva samples and rated their alertness using the Karolinska Sleepiness Scale (KSS, Akerstedt and Gillberg, 1990), a single-item, 9-point rating scale. Participants were instructed to refrain from consuming caffeinated beverages and alcohol for 24 h prior to their experimental sessions, and not to eat chocolate or bananas during the time they performed saliva sampling. There were no instructions on behavior or light exposure for the time of saliva sampling. As saliva sampling was done outside of the laboratory, it was not possible to check adherence to instructions regarding food intake. All experimental visits in the laboratory were scheduled 2 h after participants habitual wake time to avoid influence of sleep inertia during experimental sessions.

Once participants arrived in the laboratory, actimetry recordings were checked for compliance with the rest-activity-schedule based on recorded activity and light exposure. Afterwards, participants were equipped with electrodes for measurement of cardiovascular activity and an eye tracker (PupilCore, PupilLabs, Berlin, Germany; data is not part of the present manuscript).

All procedures of the experimental sessions including the cognitive task, and all ratings were implemented in a custom Python program. The experimental sessions comprised an 8-min cardiovascular baseline, a 15-min light exposure phase, and a 5-min cognitive test phase (see Fig. 1). During the cardiovascular baseline, participants were shown a movie displaying landscape recordings. During the light exposure phase, participants had the opportunity to read in magazines on an e-reader (tolino epos 2, Longshine Technologie GmbH, Ahrensburg, Germany) with the background light switched off. Baseline light condition was 1 lux and experimental lighting conditions were either 1 lux or 65 lux at eye level (distance 100 cm from the computer screen, height 120 cm), implemented through dim room light and a black or white background on the computer screen. Details on experimental light conditions and effects of light intensity are published elsewhere (preliminary study in Wüst and Lasauskaite, 2024). The cognitive task was an auditory 2-back task which consisted of 150 trials and lasted 5 min in total. Participants were presented a random series of tones of different pitches (290 Hz–590 Hz in 30 Hz steps), lasting 1 s each. When the tone presented was the same as the second last tone presented before (positive trial), participants were required to press the space bar on the computer keyboard. When the tone did not match (negative trial), no reaction of the participant was required. Participants were given 1 s to respond, before the next tone started. One third of the trials were positive, i.e., required the participant to respond. Participants responses and reaction times were recorded. Based on previous research (Lasauskaite et al., 2023; Lo et al., 2012), task difficulty was expected to be moderate.

Participants rated their alertness using the KSS before the cardiovascular baseline, after the light exposure phase, and after task performance, and reported their current mood with two positive (cheerful and happy) and two negative (sad and depressed) hedonic tones from the UWIST Mood Adjective Checklist (UMAC; Matthews et al., 1990) on a scale from 1 (not at all) to 7 (very much). After the task, participants additionally rated task perception (perceived difficulty, subjective effort, and capability) on the same 7-point Likert scale. Light perception was assessed using four items (light comfort, light intensity, light intensity preference, and glare) adapted from the German questionnaire for evaluation of lighting situations (Moosmann and Vandahl, 2015) on a scale from 1 to 7.

### 2.3 Measurements and apparatus

#### 2.3.1 Melatonin analysis

Saliva samples were taken by participants independently following instructions they received during the screening visit. Saliva was collected using Salivettes (Sarstedt, Nümbrecht, Germany). Participants were asked to refrigerate saliva samples once they were taken. Saliva sampling started 5 h prior to habitual bedtime and hourly samples were taken until participants went to bed, resulting in 6 samples per evening for NR nights and 9 samples for SR nights. In the morning, participants took two more saliva samples upon wake-up and 1 h later. Participants brought the saliva samples to the laboratory when they arrived for their experimental visits. The saliva samples were then centrifuged and stored in a freezer (-20 C). Saliva melatonin analysis was conducted using radioimmunoassays (RIA, RK-DSM2), provided through a third party (NovoLytiX GmbH, Witterswil, Switzerland). Melatonin onset for each session was determined using the hockey-stick method (Danilenko et al., 2014).

#### 2.3.2 Cardiovascular measurements

Cardiac PEP was assessed from simultaneous recording of the electrocardiogram (ECG) and the impediance cardiogram (ICG) using the CardioScreen 1000 device (medis Medizinische Messtechnik, Ilmenau, Germany). The device requires a set of four disposable electrodes, attached to the left upper body of the participant (two on the side of the neck, one at the line under the breast and one 10 cm below the third one) and a clip attached to the right earlobe. ECG and ICG were stored on a personal computer. SBP, DBP, and HR were assessed simultaneously to PEP to control for ventricular filling and arterial pressure as well as eventual effects of preload or afterload (Sherwood et al., 1990). SBP, DBP, and HR were recorded using the SOMNOtouch device (SOMNOmedics GmbH, Randersacker, Germany). The device requires four disposable electrodes attached to the upper body of the participant (below the left and right clavicle and left and right at the height of the lowest rib in a straight line down from the electrodes below the clavicles) and a finger sensor attached to the left ring or middle finger. All data was stored internally on the SOMNOtouch device and transferred to a personal computer after the experimental session.

### 2.4 Data analysis

PEP is defined as the time interval between the Q-point of the ECG and the B-point of the ICG (Berntson et al., 2004). The software “Blue Box 2” developed by Richter (2010) was used to determine PEP values (in ms) from 1-min averages of the ECG and dZ/dt of the ICG signals that were event-related to the R-peak of the ECG. R-onset was automatically detected. B-point detection was done according to the algorithm by (Lozano et al., 2007) and manually corrected. Artifact rejection was performed manually, with the scorer being unaware of the experimental conditions. Blood pressure and HR were averaged in the same 1-min intervals.

The last 4 min of the baseline period provided stable values (BF_10_ for time effect: 0.113, indicating no impact of time on PEP) and were averaged for cardiovascular baseline measures. Reactivity of cardiovascular measures was calculated as change from baseline.

**Sleep time-derived chronotypes** (early, intermediate, and late) were determined by splitting the dataset into thirds based on average sleep time as indicated in the MCTQ. Average sleep time was calculated as follows: (sleep time on workdays ∗ 5 + sleep time on free days ∗ 2)/7. We included sleep time on workdays into determining sleep time-derived chronotypes because the participants adhered to a fixed sleep-wake schedule for four nights before saliva sampling, which might have impacted melatonin onset. Furthermore, an average of work and free days mid sleep was stronger associated with melatonin onset than free days only (Kantermann and Burgess, 2017).

**Melatonin onset-derived chronotypes** (early, intermediate, and late) were determined by splitting the dataset into thirds based average melatonin onset. Average melatonin onset was calculated as the mean of melatonin onset in the NR and the SR condition, if both were available. In cases when melatonin onset was only available from one of the experimental conditions (N = 7), the available value was used for average melatonin onset. For one participant, melatonin onset could not be determined in the NR nor the SR condition.

A Bayesian approach was applied for statistical analyses using R and the packages *rstanarm* (Goodrich et al., 2020) and *bayestestR* (Makowski et al., 2019). The prior was normally distributed with a mean of 0 and a SD of 2.5. Model estimations used four chains with 20000 iterations, which was required for accurate estimation of Bayes factors. 95 %-credible intervals are reported. The sleep condition was added to statistical models, as we considered it a potentially confounding variable for the outcomes analyzed in the present analyses (see our previous publication Wüst and Lasauskaite, 2024 for sleep duration effects). The interaction of chronotypes and sleep duration was added to statistical models, as we considered it possible that chronotypes react differently to the loss of 3 h of sleep in the beginning of the night: on the one hand, later types might generally be more used to experiencing sleep restriction due to late bed times but societal factors requiring early wake times, on the other hand, earlier types might be less affected by sleep restriction due to cognitive testing in the morning occurring at their optimal time of the day. All statistical models contained random effects for participants to account for repeated measurements. As we found session effects on task performance in the primary statistical analyses, experimental session was added as a covariate to the statistical models for task performance to control for learning effects from the first to the second experimental session. Primary analyses did not show session effects for any other outcome, wherefore session was not added as a covariate to any other statistical models, keeping them as simple as possible. Model estimates are shown with early types and the 8 h sleep condition as reference categories. These model estimates provide absolute effect sizes and have the same unit as the respective outcome. Commonly, substantial differences are detected, when the credible interval does not include zero. Bayes factors indicate a comparison of likelihoods, i.e., a comparison of the model with (H1) versus without (H0) the respective predictor. For the example of PEP response predicted by sleep-time derived chronotype (section 4.4.1), this would be the comparison of the H1 model including sleep-time derived chronotype as predictor, sleep condition as potential confounding variable, the interaction of chronotype x sleep condition, and random effects for subjects [PEP response ∼ sleep-time derived chronotype + sleep condition + sleep-time derived chronotype∗sleep condition + (1|subject)] with the H0 model [PEP response ∼ sleep condition + sleep-time derived chronotype∗sleep condition + (1|subject)]. Conventions for the interpretation of Bayes factors (BF_10_) are as follows: 1 to 3.2: barely worth mentioning, 3.2 to 10: substantial, 10 to 31.6: strong, 31.6 to 100: very strong, and >100 decisive evidence *for H1* and 1 to 1/3.2: barely worth mentioning, 1/3.2 to 1/10: substantial, 1/10 to 1/31.6: strong, 1/31.6 to 1/100: very strong, and <1/100 decisive evidence *for H0* (Jeffreys, 1998).

Chronotypes were classified as early, intermediate, and late as this inherits an advantage for the statistical analyses, namely by using chronotypes as a categorical variable, we can test for differences between groups, i.e., whether intermediate or late types differ from early types, independent of the kind of mathematical association. For example, a U-shaped association could be shown by a difference between early and intermediate types while no difference between early and late types is observed. Using chronotype as a scale variable and testing linear models would only allow to test for linear associations, while any other association would require additional mathematical modelling. Given the exploratory nature of the analyses presented here, using chronotype as a categorical variable is advantageous and the results should lead future research to apply more specific statistical models.

## 3 Results

Valid MCTQ data was available for 38 participants. Using the hockey-stick method, melatonin onset could be determined for 71 of the 80 data sets (provided by 39 participants).

**Fig. 2:**
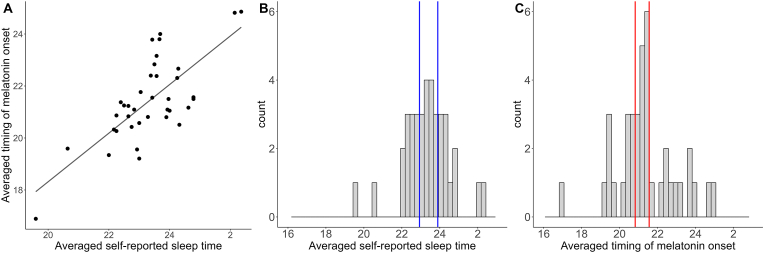
Descriptives of self-reported sleep time and timing of melatonin onset. A: Association of self-reported sleep time (averaged for work and free days) and averaged timing of melatonin onset; B: Histogram of averaged reported sleep time (binwidth 15 min), vertical lines represent cutoff values for categorization into early, intermediate, and late types; C: Histogram of averaged timing of melatonin onset (binwidth 15 min), vertical lines represent cutoff values for categorization into early, intermediate, and late types.

Averaged timing of melatonin onset and averaged reported sleep time were credibly associated (Estimate = 0.9, SD = 0.2, 95 %-CI = [0.636, 1.234], see Fig. 2A).

### 3.1 Sleep time

The histogram of averaged sleep times is depicted in Fig. 2B. Averaged sleep time was between 19:36 and 02:22, *M* = 23:22, *SD* = 86 min. Cut-off values for the split were 22:56 and 23:54. The split into sleep time-derived chronotypes resulted in 13 early types (8 women), 13 intermediate types (9 women), and 14 late types (7 women). Experimental sessions started between 6:30 and 12:30.

### 3.2 Timing of melatonin onset

Timing of melatonin onset was averaged from both sleep conditions if it was possible to determine melatonin onset for both conditions. Averaged timing of melatonin onset was between 16:53 and 00:52, *M* = 21:23, *SD* = 97 min. Melatonin onset-derived chronotypes were computed through a tercentile split of the dataset based on the average timing of the melatonin onset into early (N = 13, 9 women), intermediate (N = 13, 6 women), and late (N = 14, 9 women) types. Cut-off values were 20:58 and 22:17. The histogram of averaged melatonin onsets is depicted in Fig. 2C. Absolute differences of timing of melatonin onset between both assessments ranged from 1 min to 3 h 54 min (*M* = 1h 11 min, *SD* = 56 min) in 27 datasets with 2 values available.

### 3.3 Comparison of chronotype categorizations

Cronbach's alpha for sleep time-derived chronotypes and melatonin onset-derived chronotypes was 0.578, which can be interpreted as poor agreement between both measures. Only for 14 of 40 participants (33.6 %), the categorization of chronotypes from the MCTQ and melatonin onset was the same.

### 3.4 Sleep time-derived chronotype

#### 3.4.1 Association with cardiovascular response

Baseline values for cardiovascular measurements are presented in Supplementary Table S1. Comparisons of baseline values of PEP and SBP between sleep time-derived chronotypes were inconclusive (PEP: *BF*_*10*_ = 0.658, SBP: *BF*_*10*_ = 1.36). There was weak evidence for a higher baseline HR in late sleep-time derived chronotypes (*BF*_*10*_ = 3.77; no estimate credibly different from 0; see Supplementary Table S2).

**Fig. 3:**
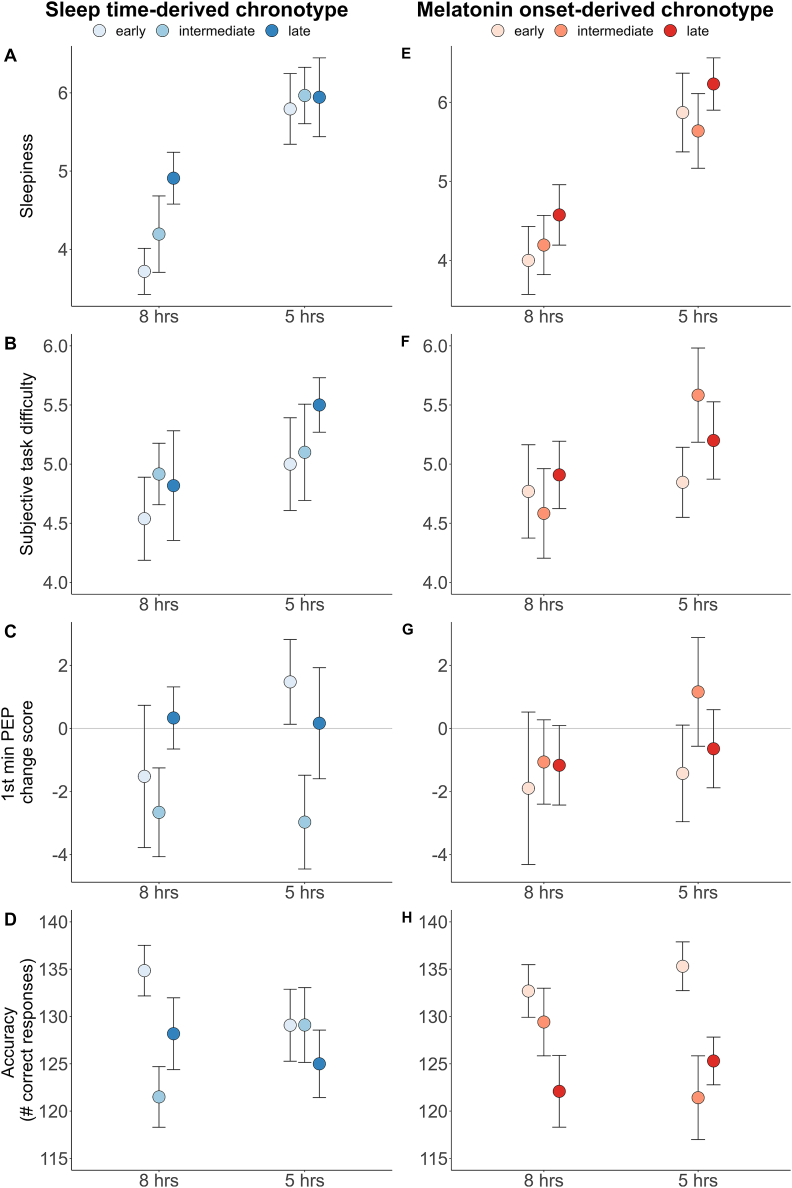
Results (means and SEs) of alertness, perceived task difficulty, PEP response, and task accuracy for sleep time-derived chronotypes (left panel, blue colors) and melatonin onset-derived chronotypes (right panel, red colors). *Note*. Sleepiness was assessed using the Karolinska Sleepiness Scale (KSS), displayed values are means over all three KSS assessments; PEP: pre-ejection period.

For PEP change score (Fig. 3C), model comparisons revealed a BF_10_ of 0.631 for the model including sleep time-derived chronotype. Individual comparisons did not reveal any substantial differences as indicated by mean estimates and 95 % credibility intervals. For SBP change score, a BF_10_ of 0.268 indicated no differences between sleep time-derived chronotypes in SBP reactivity during the 1st min of the cognitive challenge. No comparison yielded substantial differences as indicated by estimates and 95 %-CIs. For HR change score, model comparisons revealed a BF_10_ of 0.139, indicating no differences in HR reactivity between sleep time-derived chronotypes. Accordingly, estimates and 95 %-CIs did not show any substantial differences. Estimates, SDs, and 95 % credibility intervals (CIs) are shown in Table 1, means and SDs for cardiovascular reactivity are presented in Supplementary Table S3.

**Table 1:** Statistical models for associations between sleep time-derived chronotypes and cardiovascular response.

			Estimate (M)	SD	95 %-CI
PEP	Sleep time-derived chronotype	intermediate	−1.3	1.6	−4.279, 1.829
		late	1.1	1.6	−2.106, 4.264
	Sleep	5 h	1.3	1.2	−1.001, 3.556
	Sleep time-derived chronotype x sleep	intermediate x 5 h	−1.2	1.7	−4.369, 2.122
		late x 5 h	−1.6	1.7	−4.863, 1.854
SBP	Sleep time-derived chronotype	intermediate	0.9	1.4	−1.805, 3.654
		late	−1.1	1.4	−3.822, 1.669
	Sleep	5 h	−0.8	0.9	−2.655, 1.026
	Sleep time-derived chronotype x sleep	intermediate x 5 h	1.0	1.3	−1.534, 3.447
		late x 5 h	1.1	1.3	−1.403, 3.575
HR	Sleep time-derived chronotype	intermediate	−0.3	1.3	−2.727, 2.212
		late	−1.0	1.3	−3.453, 1.565
	Sleep	5 h	−1.1	1.1	−3.211, 1.074
	Sleep time-derived chronotype x sleep	intermediate x 5 h	−0.1	1.5	−3.110, 2.833
		late x 5 h	−0.7	1.5	−3.637, 2.247

*Note*. PEP: pre-ejection period, SBP: systolic blood pressure, HR: heart rate, 95 %-CI: 95 % credible interval.

#### 3.4.2 Association with task performance

Statistical models for task performance are displayed in Table 2. Model comparisons revealed a BF_10_ of 0.814, being inconclusive regarding the effect of sleep time-derived chronotype on task performance (Fig. 3D). Substantial differences were only found between experimental sessions, but not between sleep-time derived chronotypes, sleep conditions, nor for the interaction.

**Table 2:** Statistical models for associations between sleep time-derived chronotype and task performance.

		Estimate (M)	SD	95 % CI
Sleep time-derived chronotype	intermediate	−1.8	2.1	−5.968, 2.430
	late	−0.9	2.1	−4.998, 3.340
Sleep	5 h	−0.7	1.5	−3.694, 2.329
Sleep time-derived chronotype x sleep	intermediate x 5 h	1.2	2.0	−2.797, 5.075
	late x 5 h	−0.6	2.0	−4.534, 3.223
S**ession**	**2nd**	**5.6**	**1.5**	**2.655, 8.411**

#### 3.4.3 Association with self-report measures

##### 3.4.3.1 Association with alertness

For alertness (Fig. 3A), assessed as sleepiness rating in the KSS, model comparison revealed a BF_10_ of 0.019, indicating no difference in alertness between sleep time-derived chronotypes. Nevertheless, the model estimate for the interaction of late type x SR was credibly different from zero, indicating that late types in the 5 h sleep condition report lower sleepiness compared to early types in the 8h sleep condition (0.9 on the 7-point scale). The statistical model is displayed in Table 3, cell means and SDs are shown in Supplementary Table S4.

**Table 3:** Statistical model for associations of sleep time-derived chronotype and alertness.

		Estimate (M)	SD	95 % CI
Sleep time-derived chronotype	intermediate	0.4	0.6	−0.754, 1.526
	late	1.1	0.6	−0.072, 2.175
S**leep**	**5h**	**2.0**	**0.3**	**1.409, 2.615**
Sleep time-derived chronotype x sleep	intermediate x 5h	−0.2	0.5	−1.151, 0.677
	**late x 5h**	**−0.9**	**0.5**	**−1.838, -0.021**
**KSS measurement**		**0.6**	**0.1**	**0.339, 0.802**

Note. Credible differences are marked in bold. KSS: Karolinska Sleepiness Scale (Akerstedt and Gillberg, 1990).

##### 3.4.3.2 Association with task ratings

For perceived task difficulty (Fig. 3B), model comparison revealed a BF_10_ of 0.008, indicating no effect of sleep time-derived chronotype on perceived difficulty. Consequently, no estimate was substantially different from zero. For subjective effort, model comparisons revealed a BF_10_ of 0.060, indicating no effect of sleep time-derived chronotype on subjective effort. The estimate for intermediate comapred to early types differed from zero, none of the other estimates was substantially different from zero. For subjective capability, model comparisons revealed a BF_10_ of 2.76E-4, indicating no effect of sleep time-derived chronotype on capability. Consequently, no estimate was credibly different from zero. Statistical models are shown in Table 4. All cell means and SDs are presented in Supplementary Table S5.

**Table 4:** Statistical models for associations of sleep time-derived chronotypes with subjective task ratings.

			Estimate (M)	SD	95 % CI
Perceived difficulty	Sleep time-derived chronotype	intermediate	0.3	0.5	−0.592, 1.283
		late	0.2	0.5	−0.726, 1.182
	Sleep	5 h	0.4	0.4	−0.340, 1.226
	Sleep time-derived chronotype x sleep	intermediate x 5 h	−0.2	0.6	−1.400, 0.926
		late x 5 h	0.3	0.6	−0.885, 1.416
Subjective effort	Sleep time-derived chronotype	**intermediate**	**1.0**	**0.5**	**0.009, 1.940**
		late	0.4	0.5	−0.582, 1.389
	Sleep	5 h	0.6	0.4	−0.244, 1.358
	Sleep time-derived chronotype x sleep	intermediate x 5 h	−0.6	0.6	−1.797, 0.593
		late x 5 h	−0.2	0.6	−1.362, 0.994
Subjective capability	Sleep time-derived chronotype	intermediate	−0.7	0.5	−1.744, 0.346
		late	0.0	0.5	−1.063, 1.040
	Sleep	5 h	−0.4	0.4	−1.085, 0.370
	Sleep time-derived chronotype x sleep	intermediate x 5 h	0.8	0.5	−0.281, 1.868
		late x 5 h	−0.4	0.5	−1.529, 0.629

### 3.5 Melatonin onset-derived chronotype

#### 3.5.1 Association with cardiovascular response

Model comparisons revealed inconclusive Bayes factors for differences between melatonin onset-derived chronotypes for baseline PEP (BF_10_ = 2.07), SBP (BF_10_ = 0.72), and HR (BF_10_ = 1.63). Means and SDs of cardiovascular baseline measurements are shown in supplementary Table S6.

For cardiac PEP change score (Fig. 3G), the model comparison revealed a BF_10_ of 0.232, indicating that melatonin onset-derived chronotypes did not differ in PEP change score. Consequently, no model estimate was credibly different from zero. For SBP change score, model comparison revealed a BF_10_ of 0.263, indicating no impact of melatonin onset-derived chronotype on SBP change score. No model estimate was credibly different from zero. For HR change score, the model comparison showed a BF_10_ of 0.595, being inconclusive regarding the effect of melatonin onset-derived chronotype on HR reactivity. None of the model estimates was credibly different from zero. All model estimates with corresponding SDs and 95 % CIs are displayed in Table 5. Cell means and SDs are presented in Supplememtary Table S7.

**Table 5:** Statistical models for melatonin-type effects on physiological response.

			Estimate (M)	SD	95 % CI
PEP	Melatonin onset-derived chronotype	intermediate	0.7	1.6	−2.389, 3.709
		late	0.3	1.6	−2.970, 3.469
	Sleep	5h	0.4	1.2	−2.019, 2.797
	Melatonin onset-derived chronotype x sleep	intermediate x 5h	0.9	1.6	−2.207, 4.133
		late x 5h	−0.1	1.7	−3.582, 3.320
SBP	Melatonin onset-derived chronotype	intermediate	−2.2	1.4	−4.888, 0.592
		late	−1.4	1.4	−4.115, 1.370
	Sleep	5h	−0.3	0.9	−2.196, 1.526
	Melatonin onset-derived chronotype x sleep	intermediate x 5h	0.3	1.3	−2.196, 2.816
		late x 5h	0.0	1.3	−2.613, 2.508
HR	Melatonin onset-derived chronotype	intermediate	0.0	1.2	−2.387, 2.504
		late	−1.4	1.3	−3.829, 1.141
	Sleep	5h	−0.5	1.1	−2.593, 1.647
	Melatonin onset-derived chronotype x sleep	intermediate x 5h	−2.2	1.5	−5.066, 0.678
		late x 5h	−0.9	1.5	−3.800, 2.083

*Note*. PEP: pre-ejection period, SBP: systolic blood pressure, HR: heart rate, 95 %-CI: 95 % credible interval.

#### 3.5.2 Association with task performance

Model comparisons showed a BF_10_ of 4.23 for task performance (Fig. 3H), when controlling for experimental session. The Bayes factor indicates evidence for an effect of melatonin onset-derived chronotype on task performance. Yet, no model estimate was credibly different from zero, indicating an overall bad fit of the statistical models and high variance in task performance, which cannot be explained by any of the predictors included in the statistical models. The statistical model for task performance including model estimates with their corresponding SDs and 95 % CIs is displayed in Table 6.

**Table 6:** Statistical model for the associations of melatonin onset-derived chronotypes and task performance.

		Estimate (M)	SD	95 % CI
melatonin onset-derived chronotype	intermediate	−1.1	2.1	−5.177, 3.036
	late	−2.6	2.2	−6.750, 1.664
sleep	5h	0.2	1.5	−2.651, 3.103
melatonin onset-derived chronotype x sleep	intermediate x 5h	−3.3	2.0	−7.108, 0.528
	late x 5h	−0.2	2.0	−4.067, 3.638
**session**	**2nd**	**5.8**	**1.4**	**2.953, 8.412**

#### 3.5.3 Association with self-report measures

##### 3.5.3.1 Association with alertness

The model comparison showed a BF_10_ of 0.003 for melatonin onset, indicating that melatonin onset-derived chronotype does not affect alertness ratings (Fig. 3E). Consequently, none of the model estimates for melatonin onset-derived chronotype was credibly different from zero. Though, model estimates indicate substantial effects of SR and time point of alertness measurement, reflecting an increase in sleepiness over time. All model estimates with corresponding SDs and 95 % CIs are displayed in Table 7, cell means and SD are shown in Supplementary Table S8.

**Table 7:** Associations of melatonin onset-derived chronotypes and alertness.

		Estimate (M)	SD	95 % CI
Melatonin onset-derived chronotype	intermediate	0.2	0.6	−0.892, 1.389
	late	0.4	0.6	−0.779, 1.557
S**leep**	**5h**	**1.8**	**0.3**	**1.205, 2.433**
Melatonin onset-derived chronotype x sleep	intermediate x 5h	−0.4	0.5	−1.317, 0.477
	late x 5h	−0.1	0.5	−1.020, 0.862
**KSS measurement**		**0.6**	**0.1**	**0.334, 0.801**

*Note*. KSS: Karolinska Sleepiness Scale (Akerstedt and Gillberg, 1990).

##### 3.5.3.2 Association with task ratings

For subjective task difficulty (Fig. 3F), model comparison revealed a BF_10_ of 0.006, indicating that melatonin onset-derived chronotype does not affect perceived task difficulty. Consequently, no model estimate was credibly different from zero. For subjective effort, the model comparison revealed a BF_10_ of 0.002, indicating no effect of melatonin onset-derived chronotype on subjective effort. Consequently, none of the model estimates was credibly different from zero. For subjective capability, model comparison revealed a BF_10_ of 0.125, indicating no effect of melatonin onset-derived chronotype on capability. Nevertheless, the estimate of the interaction of intermediate types x SR was credibly different from zero. Full statistical models for task ratings are presented in Table 8, cell means and SDs are presented in Supplementray Table S9.

**Table 8:** Statistical models for associations of melatonin onset-derived chronotypes and task ratings.

			Estimate (M)	SD	95 % CI
Perceived difficulty	Melatonin-type	intermediate	−0.2	0.5	−1.110, 0.763
		late	0.1	0.5	−0.816, 1.105
	Sleep	5h	0.1	0.4	−0.669, 0.866
	Melatonin-type x sleep	intermediate x 5h	0.9	0.6	−0.210, 1.998
		late x 5h	0.2	0.6	−0.961, 1.345
Subjective effort	Melatonin-type	intermediate	−0.3	0.5	−1.326, 0.666
		late	0.0	0.5	−1.034, 0.982
	Sleep	5h	0.3	0.4	−0.518, 1.123
	Melatonin-type x sleep	intermediate x 5h	0.1	0.6	−1.119, 1.262
		late x 5h	−0.1	0.6	−1.320, 1.129
Subjective capability	Melatonin-type	intermediate	0.5	0.5	−0.538, 1.521
		late	−0.6	0.5	−1.687, 0.412
	Sleep	5h	−0.1	0.4	−0.768, 0.605
	Melatonin-type x sleep	**intermediate x 5h**	**−1.0**	**0.5**	**−2.014, -0.006**
		late x 5h	0.6	0.5	−0.466, 1.636

## 4 Discussion

The present work explored the association of chronotypes derived from sleep timing and melatonin onset with mental effort. The study design included one normally rested and one sleep restricted condition per participant, and the results showed that alertness was lower after sleep restriction, but perceived task demand and mental effort remained unaffected by sleep restriction. In our study, we found no differences between chronotypes in alertness, perceived task difficulty, or effort-related cardiovascular response during a cognitive challenge presented in the morning, regardless of whether chronotypes were derived from sleep time or timing of melatonin onset.

Contrary to our expectations, participants with different chronotypes did not experience task demand differently and therefore did not differ in the amount of effort they invested to perform a given task in the morning. This was true for both, self-reported sleep-time derived as well as melatonin onset-derived chronotypes. Based on the findings by Carbajal and colleagues (2019), who showed that mental effort and associated cardiovascular response increased at the non-optimal time for performance, i.e., in the afternoon for morning types, in women. They called this phenomenon a circadian mismatch, where early and late chronotypes perform at non-optimal times, as morning types are alert early and struggle with fatigue later, while late types rather struggle with fatigue early and are more alert later during the day. Based on this reasoning and findings, one would expect early types to exert less effort in the morning compared to late types. In our study though, effort-related cardiovascular response, as measured by cardiac PEP and SBP reactivity did not differ for sleep time-derived nor for melatonin onset-derived chronotypes. By testing all participants at pre-defined times in the morning and in the afternoon, it could be speculated whether Carbajal and colleagues rather observed associations of circadian timing and mental effort. Certainly, circadian timing should be expected to be associated with chronotypes and would therefore be expected to yield similar effects. In our study, we adjusted the time of cognitive testing to habitual sleep time and this way minimized variability in circadian timing between participants in our study. Nevertheless, our data indicates that there might be no difference in invested effort between chronotypes, when a challenge is scheduled at a similar circadian time adjusted for chronotype. Finding relatively wide credible intervals which include zero might indicate that in future studies, it could be beneficial to include a larger sample, allowing for a more precise computation of model estimates. Wide credible intervals could also indicate differences in participants' ability to adapt to morning challenges, especially after shortened sleep duration. Potentially, individuals who experience shortened sleep frequently or regularly due to their lifestyle, might be able to adapt better to cognitive challenges in the morning. In our study, we used a 2-back working memory paradigm as the cognitive challenge, which we expected to be difficult but still feasible in the sleep restricted condition, based on previous results. Of course, task difficulty perception is individual and differences in subjective perception of task difficulty might have led to wide ranges in the observed cardiovascular response to the cognitive challenge. Future investigations might benefit from extensive pretests to determine the optimal task difficulty level to achieve low to medium task difficulty in the normally-rested condition and high but still feasible task difficulty in the sleep-restricted condition. Additionally, future studies could use another experimental procedure, which does not require the participant to slightly move while grabbing reading material, even though this did not cause any measurement errors in previous studies conducted in our lab (Lasauskaite et al., 2019, 2023; Lasauskaite and Cajochen, 2018). Our results indicate that differences in effort investment might not be the mechanism explaining differences in cognitive performance between early and late chronotypes. Importantly, Bayesian models revealed evidence for no differences between chronotypes in cardiovascular response, which goes beyond absence of evidence from a frequentist statistical analysis.

Although chronotype is a continuous variable that tends to follow a normal distribution (Roenneberg et al., 2007), we propose a resource-efficient approach to our exploratory analysis. Instead of keeping sleep timing and timing of melatonin onset, respectively, as scale variables, we categorized our participants as early, intermediate, and late chronotypes. This allowed us to form three equally sized groups based on chronotype. Had we kept sleep timing and time of melatonin onset as scale variables, most of the participants would have clustered around the mean times, with fewer at the extremes. Regarding statistical analyses, this might have been a disadvantage. However, creating chronotype groups and performing statistical tests with these groups as predictors allowed group differences to be identified without the need to specify the association between scale variables a priori (e.g., linear, quadratic, or exponential). This procedure was chosen because it is a resource efficient approach for an explorative analysis. Future studies should take into account the results of the present study and might employ more specific statistical models.

Furthermore, our study was conducted between November 2020 and July 2021, when several restrictions were in place in Switzerland due to the COVID-19 pandemic. We did not inquire our participants to what extent their daily schedules and the whole daily or weekly rhythm was corresponding to the times without restrictions. We can speculate that with less obligations outside home, mostly remote online work and university classes, and less opportunities for socializing, participants could have been quite aligned with their chronotype. Without pandemic restrictions, this synchronization might be challenging due to social obligations, and so late types still have to get up early, for instance, for university or work, and thus sleep schedules would be farther from preferred bed times compared to during the pandemic. As we adapted the timing of experimental sessions to the sleep schedules, consequently, conducting the study during the pandemic might have led to experimental sessions taking place closer to preferred times of the day than outside of the pandemic. Thus, the temporal match between the timing of the cognitive task and the preferred time of day for diurnal activity may have resulted in optimal morning performance for either chronotypes.

Our study did not reveal credible evidence for an association between chronotype and task performance in the morning, when we adapted the time of cognitive testing to habitual wake time. Other studies that adapted the timing of cognitive tests to individual sleep schedules likewise did not find time of day dependent differences in cognitive performance between chronotypes (Mongrain et al., 2008; Schmidt et al., 2015). Maierova and colleagues also scheduled test times according to individual circadian time and found associations between performance and chronotypes interacting with light conditions, i.e., late but not early types performed better under bright compared to dim light during a working memory paradigm, but no association with chronotype for sustained attention was shown (Maierova et al., 2016). Contrary results found chronotype differences in cognitive performance, all using pre-defined schedules for cognitive testing, which were independent of habitual sleep timing (Horne et al., 1980; Lara et al., 2014; Martínez-Pérez et al., 2020; Taillard et al., 2011). Potentially, differences found between chronotypes might reflect differences in circadian timing, which cannot be observed when the time of cognitive testing is adapted to habitual sleep timing, as we did in our study. Further, it is possible that we did not observe associations between chronotype and mental effort and task performance because our study included young, healthy participants who generally performed at high levels.

Self-reported sleep time and timing of melatonin onset were associated in our study. However, we observed poor agreement between sleep time-derived and melatonin onset-derived chronotype categorization, despite the association between self-reported sleep time and timing of melatonin onset. This finding aligns with results from earlier studies finding poor concordance between chronotypes derived from different sources (Reiter et al., 2021). The differences in chronotype categorization based on sleep timing and timing of melatonin onset highlight the importance of a careful, theory-driven selection of chronotype measures in future studies.

### 4.1 Strengths

Substantial parts of our study protocol were conducted in an ambulatory fashion, including saliva sampling, a fixed sleep-wake-schedule, and a manipulation of sleep-duration. Even though this might have biased the data to some extent, it also greatly increases the generalizability of our results. Participants only received minimal instructions on behavior during saliva sampling, i.e., participants were instructed to refrain from certain foods and beverages which are known to affect determined salivary melatonin concentrations, but were not limited in their behavior regarding light exposure or activity. Therefore, the melatonin onsets measured may not reflect the participants' circadian phase as accurately as a DLMO assessed in the laboratory, but they do represent the participants' evening melatonin onset in their habitual environment, which could be called real-life melatonin onset. The association of melatonin onset with self-reported sleep time indicated, that timings of melatonin onset assessed in the field indeed reflect circadian time. This might be important for future studies but also for clinical assessments of melatonin in patients with disorders affecting their circadian system.

For the first time, we have analyzed differences in mental effort between chronotypes determined from a biological marker, i.e., timing of melatonin onset, that might be more objective compared to self-reports. We were further able to compare melatonin onset-derived with sleep time-derived chronotypes, which showed only low agreement with each other. This might indicate biases in self-reports but could also be a consequence of social constraints determining sleep time.

### 4.2 Limitations

The included sample of participants is not balanced for chronotypes, therefore the range in which participants were categorized as intermediate types is rather small for sleep time-derived and melatonin onset-derived chronotypes. Furthermore, the sample size may need to be adjusted to achieve greater statistical power and allow for more specific model estimations. Also, in future studies a second cognitive challenge might be presented at a later time of day, i.e., in the afternoon, to expose late chronotypes to the cognitive challenge at a potentially more optimal circadian time. Additionally, 13 or 14 participants per group are small group sizes, limiting the precision of parameter estimation, which is also reflected in rather wide credible intervals. Therefore, future studies might not only benefit from an improved experimental design but also from larger sample sizes.

Home assessment of saliva sampling might have led to a delay in assessed timing of melatonin onset (Moderie et al., 2020; Pullman et al., 2012), yet it can still be seen as a valid estimate of circadian timing. In our study, we needed to rely on the participants adhering to instructions on the timings of saliva samples and we have no knowledge on participants behavior, including food and drinks, during the period of saliva sampling.

By adjusting the timing of experimental sessions to participants habitual wake times, we intended to minimize variation in circadian timing of cognitive testing between participants. Yet, due to obligations such as university or work schedules, participants were not able to freely select their rest-activity-schedule to their personal preferences. The fixed sleep-wake-schedule was possibly closer to the participants usual sleep times on work days than on free days. Therefore, circadian timing of the cognitive challenge might still have differed between participants depending on the difference between their habitual sleep times on work and free days, and the selected sleep times for the ambulatory phase of the study.

Our study was conducted between November 2020 and July 2021, when several restrictions were in place in Switzerland due to the COVID-19 pandemic. The restrictions allowed to continue our study and, as mentioned above, possibly allowed for daily schedules to be aligned better with an individual's chronotype. On the other hand, it possibly influenced participants activities and habitual sleep behavior, resulting in reduced social jetlag (Blume et al., 2020). Repeating the study during a time without pandemic regulations in place might reveal differential effects regarding the impact of sleep-time derived chronotypes on mental effort and reflect the phenomenon of circadian mismatch, as discussed by Carbajal and colleagues (Carbajal et al., 2019).

### 4.3 Conclusion

In the present paper, we observed poor agreement between sleep time-derived and melatonin onset-derived chronotype categorization, despite a significant association between self-reported sleep time and timing of melatonin. Melatonin onset was determined in participants’ habitual environment with no other instructions than adhering to sleep times. Furthermore, we presented exploratory evidence that chronotypes, determined from self-reported sleep times and from timing of melatonin onset is not associated with mental effort during a cognitive challenge in the morning.

## CRediT authorship contribution statement

**Larissa N. Wüst:** Writing – review & editing, Writing – original draft, Software, Methodology, Investigation, Formal analysis, Data curation. **Christian Cajochen:** Writing – review & editing, Supervision, Methodology, Conceptualization. **Ruta Lasauskaite:** Writing – review & editing, Supervision, Project administration, Methodology, Funding acquisition, Conceptualization.

## Data availability statement

The data of this article can be found at https://doi.org/10.5281/zenodo.11503187.

## Funding

This research was supported by the 10.13039/100000001Swiss National Science Foundation (Grant No. PZ00P1_179953 awarded to Dr. Ruta Lasauskaite).

## Declaration of competing interest

The authors declare the following financial interests/personal relationships which may be considered as potential competing interests: Ruta Lasauskaite reports financial support was provided by 10.13039/100000001Swiss National Science Foundation. Christian Cajochen reports a relationship with Novartis that includes: consulting or advisory. Christian Cajochen reports a relationship with Roche that includes: consulting or advisory. Christian Cajochen reports a relationship with Velux Foundation that includes: consulting or advisory and travel reimbursement. If there are other authors, they declare that they have no known competing financial interests or personal relationships that could have appeared to influence the work reported in this paper.
