# Supplementary material for: No association between chronotype and cardiovascular response to a cognitive challenge in the morning using a Bayesian approach
Source: Neurobiol Sleep Circadian Rhythms. 2025 May 9;18:100125. doi: 10.1016/j.nbscr.2025.100125 (PMC12148713; doi:10.1016/j.nbscr.2025.100125)
Supplement: Multimedia component 1 [file mmc1.pdf]

Supplementary material: No association between chronotype and cardiovascular response to a cognitive challenge in the morning using a Bayesian approach

Supplementary table S 1: Means and SDs (in brackets) for cardiovascular baseline values for sleep time-derived chronotypes

	early	intermediate	late
PEP	104.56 (8.36) (N = 24)	106.05 (8.51) (N = 19)	104.08 (9.07) (N = 18)
SBP	107.10 (11.58) (N = 23)	110.61 (11.73) (N = 21)	116.62 (11.43) (N = 23)
HR	67.36 (8.66) (N = 25)	70.57 (9.05) (N = 22)	78.30 (9.50) (N = 23)

PEP: pre-ejection period (in ms), SBP: systolic blood pressure (in mmHg), HR: heart rate (in beats per min)

Supplementary table S 2: Bayesian model for HR baseline differences

		Estimate (M)	SD	95 %-credible interval
sleep time-derived chronotype	intermediate	-0.3	2.0	-4.225, 3.639
	late	3.7	2.1	-0.368, 7.664

Supplementary table S 3: Cell means and standard deviations (in brackets) for cardiovascular reactivity in sleep time-derived chronotypes

Sleep time-derived chronotype	early		intermediate		late	
Sleep condition	5 h	8 h	5 h	8 h	5 h	8 h
PEP	1.48 (4.46) (N = 11)	-1.52 (8.13) (N = 13)	-2.97 (4.20) (N = 8)	-2.66 (4.67) (N = 11)	0.17 (5.28) (N = 9)	0.33 (2.95) (N = 9)
SBP	0.00 (4.12) (N = 11)	1.86 (5.93) (N = 9)	2.52 (4.52) (N = 10)	2.33 (4.56) (N = 11)	0.24 (3.33) (N = 12)	-0.31 (3.31) (N = 10)
HR	-1.43 (3.15) (N = 12)	0.01 (3.00) (N = 12)	-1.74 (3.35) (N = 10)	-0.58 (6.73) (N = 12)	-3.22 (3.45) (N = 12)	-1.37 (3.07) (N = 10)

PEP: pre-ejection period (in ms), SBP: systolic blood pressure (in mmHg), HR: heart rate (in beats per min)

Supplementary table S 4: Cell means and standard deviations (in brackets) for alertness ratings in sleep time-derived chronotypes

Sleep time-derived chronotype	early		intermediate		late	
Sleep condition	5 h (N = 13)	8 h (N = 13)	5 h (N = 10)	8 h (N = 12)	5 h (N = 12)	8 h (N = 11)
KSS1	5.15 (1.82)	2.69 (1.11)	5.00 (1.94)	3.50 (1.88)	5.25 (2.14)	3.36 (1.50)
KSS2	6.31 (1.93)	4.69 (1.55)	6.60 (1.58)	4.67 (2.06)	6.58 (1.98)	5.82 (1.47)
KSS3	5.92 (1.75)	3.77 (1.42)	6.30 (2.11)	4.42 (2.27)	6.00 (2.04)	5.55 (1.69)

KSS: Karolinska Sleepiness Scale (Akerstedt & Gillberg, 1990)

Supplementary table S 5: Cell means and standard deviations (in brackets) for task ratings in sleep time-derived chronotypes

Sleep time-derived chronotype	early		intermediate		late	
Sleep condition	5 h (N = 13)	8 h (N = 13)	5 h (N = 10)	8 h (N = 12)	5 h (N = 12)	8 h (N = 11)
Difficulty	5.00 (1.41)	4.54 (1.27)	5.10 (1.29)	4.92 (0.90)	5.50 (0.80)	4.82 (1.54)
Effort	5.31 (1.49)	4.69 (0.95)	5.70 (1.64)	5.75 (0.87)	5.50 (1.00)	5.18 (1.47)
Capability	3.54 (1.39)	3.92 (1.38)	3.70 (1.89)	3.17 (1.27)	3.08 (1.24)	3.91 (0.83)

Supplementary table S 6: Cardiovascular baseline means and SDs (in brackets) for melatonin onset-derived chronotypes

	early	intermediate	late
PEP	108.45 (6.75((N = 22)	100.96 (8.49) (N = 23)	105.62 (8.82) (N = 16)
SBP	111.53 (13.55) (N = 23)	111.51 (11.18) (N = 24)	111.35 (11.97) (N = 20)
HR	66.63 (8.52) (N = 25)	73.03 (8.64) (N = 24)	77.09 (10.61) (N = 21)

PEP: pre-ejection period (in ms), SBP: systolic blood pressure (in mmHg), HR: heart rate (in beats per min)

Supplementary table S 7: Cell means and standard deviations (in brackets) for cardiovascular reactivity in melatonin onset-derived chronotypes

Sleep time-derived chronotype	early		intermediate		late	
Sleep condition	5 h	8 h	5 h	8 h	5 h	8 h
PEP	-1.43 (4.85) (N = 10)	-1.90 (8.37) (N = 12)	1.16 (5.71) (N = 11)	-1.06 (4.63) (N = 12)	-0.64 (3.28) (N = 7)	-1.17 (3.78) (N = 9)
SBP	2.50 (4.49) (N = 11)	3.71 (5.80) (N = 10)	-0.15 (3.62) (N = 12)	-0.39 (3.14) (N = 10)	0.24 (3.71) (N = 10)	0.61 (4.02) (N = 10)
HR	-0.21 (3.30) (N = 12)	-0.51 (6.43) (N = 11)	0.28 (2.58) (N = 12)	-3.25 (1.88) (N = 12)	-1.67 (4.29) (N = 11)	-3.16 (3.84) (N = 10)

PEP: pre-ejection period (in ms), SBP: systolic blood pressure (in mmHg), HR: heart rate (in beats per min)

Supplementary table S 8: Cell means and standard deviations (in brackets) for alertness ratings in melatonin onset-derived chronotypes

Sleep time-derived chronotype	early		intermediate		late	
Sleep condition	5 h (N = 13)	8 h (N = 13)	5 h (N = 12)	8 h (N = 12)	5 h (N = 10)	8 h (N = 11)
KSS1	5.46 (1.71)	2.77 (1.64)	5.00 (2.09)	3.08 (1.08)	4.90 (2.08)	3.73 (1.74)
KSS2	6.54 (2.03)	5.31 (2.02)	6.17 (2.12)	5.08 (1.68)	6.80 (1.03)	4.64 (1.57)
KSS3	5.62 (2.29)	3.92 (1.98)	5.75 (1.60)	4.42 (1.56)	7.00 (1.49)	5.36 (2.06)

KSS: Karolinska Sleepiness Scale (Akerstedt & Gillberg, 1990)

Supplementary table S 9: Cell means and standard deviations (in brackets) for task ratings in melatonin onset-derived chronotypes

Sleep time-derived chronotype	early		intermediate		late	
Sleep condition	5 h (N = 13)	8 h (N = 13)	5 h (N = 12)	8 h (N = 12)	5 h (N = 10)	8 h (N = 11)
Difficulty	4.85 (1.07)	4.77 (1.42)	5.58 (1.38)	4.58 (1.31)	5.20 (1.03)	4.91 (0.94)
Effort	5.62 (1.26)	5.31 (1.44)	5.33 (1.44)	5.00 (1.21)	5.50 (1.51)	5.27(0.79)
Capability	3.62 (1.45)	3.69 (1.49)	3.08 (1.44)	4.25 (0.62)	3.60 (1.65)	3.00 (1.10)

Influence of evening light exposure on melatonin onset

Average light intensity was determined by averaging all light intensity values from 3 h prior to habitual sleep time until participants went to bed. Further, time spent in below 10 lux, between 10 and 250 lux, and above 250 lux during 3 h before habitual sleep time until participants went to bed were determined by counting the minutes of light intensity in the specific ranges. The cut off values for light intensity binning were chosen based on common light exposure recommendations (Brown et al., 2022). We analyzed light exposure from 3 h prior to habitual sleep time, as participants reported being at work or commuting before that time and had no opportunity to choose their environmental light conditions. From 3h prior to habitual sleep time we expected most participants to be in a situation with self-chosen light conditions.

Supplementary table S 10: Statistical models for effects of light intensity on melatonin onset

	Estimate (SD) [95%-CI]	Bayes Factor BF_{10}
Average light intensity	0.0 (0.0) [-0.009, 0.010]	1.04E-05
Time spent in 10 to 250 lux	0.007 (0.003) [0.001, 0.013]	51.08
Time spent in above 250 lux	-0.044 (0.029) [-0.103, 0.012]	27.56

Average light intensity from 3 h to prior to habitual sleep time until scheduled sleep time measured at the wrist did not have any substantial impact on melatonin onset. Time spent in above 250 lux had a substantial impact on melatonin onset. Contrary to our expectations time spent above 250 lux was associated with an earlier melatonin onset. As expected, time spent in 10 to 250 lux substantially delayed melatonin onset. The estimate indicated that each minute spent in 10 to 250 lux delayed melatonin onset by 0.007 hours, corresponding to 25 seconds. The full statistical models are displayed in Supplementary table S 10.
